# Supplementary material for: DMRforPairs: identifying Differentially Methylated Regions between unique samples using array based methylation profiles
Source: BMC Bioinformatics. 2014 May 15;15:141. doi: 10.1186/1471-2105-15-141 (PMC4046028; doi:10.1186/1471-2105-15-141)
Supplement: Additional file 2 — DMRforPairs output for the comparison of A431-MCF7 and NA17018-NA17105. Please start from the HTML files in each folder. Available via the BMC Bioinformatics website. [file 1471-2105-15-141-S2.zip › 1394847754114233_MOESM2_ESM/A431_MCF7/figures/36802.pdf]

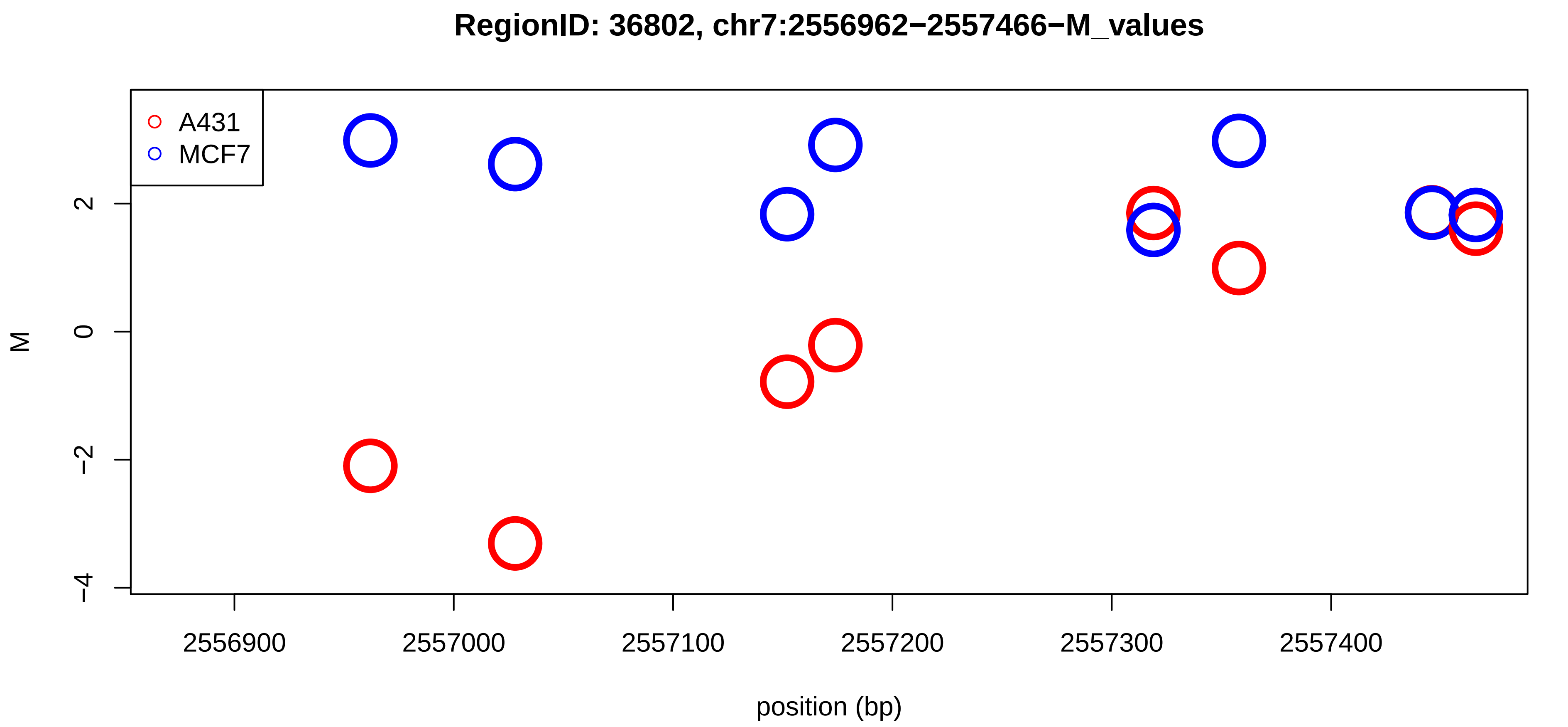

RegionID: 36802, chr7:2556962−2557466−M_values
A431
MCF7
M
−4
−2
0
2
2556900
2557000
2557100
2557200
2557300
2557400
position (bp)


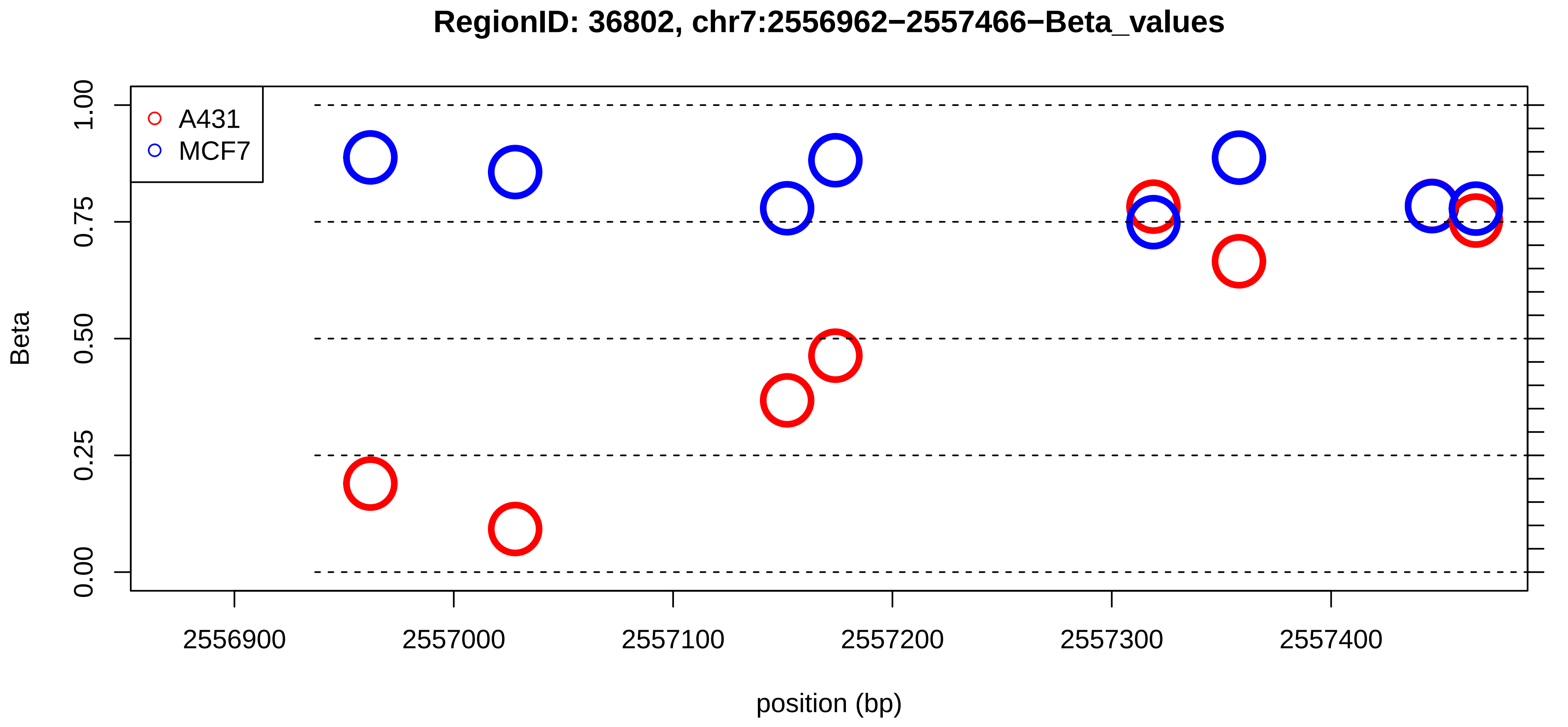

RegionID: 36802, chr7:2556962−2557466−Beta_values
A431
MCF7
Beta
0.00
0.25
0.50
0.75
1.00
2556900
2557000
2557100
2557200
2557300
2557400
position (bp)

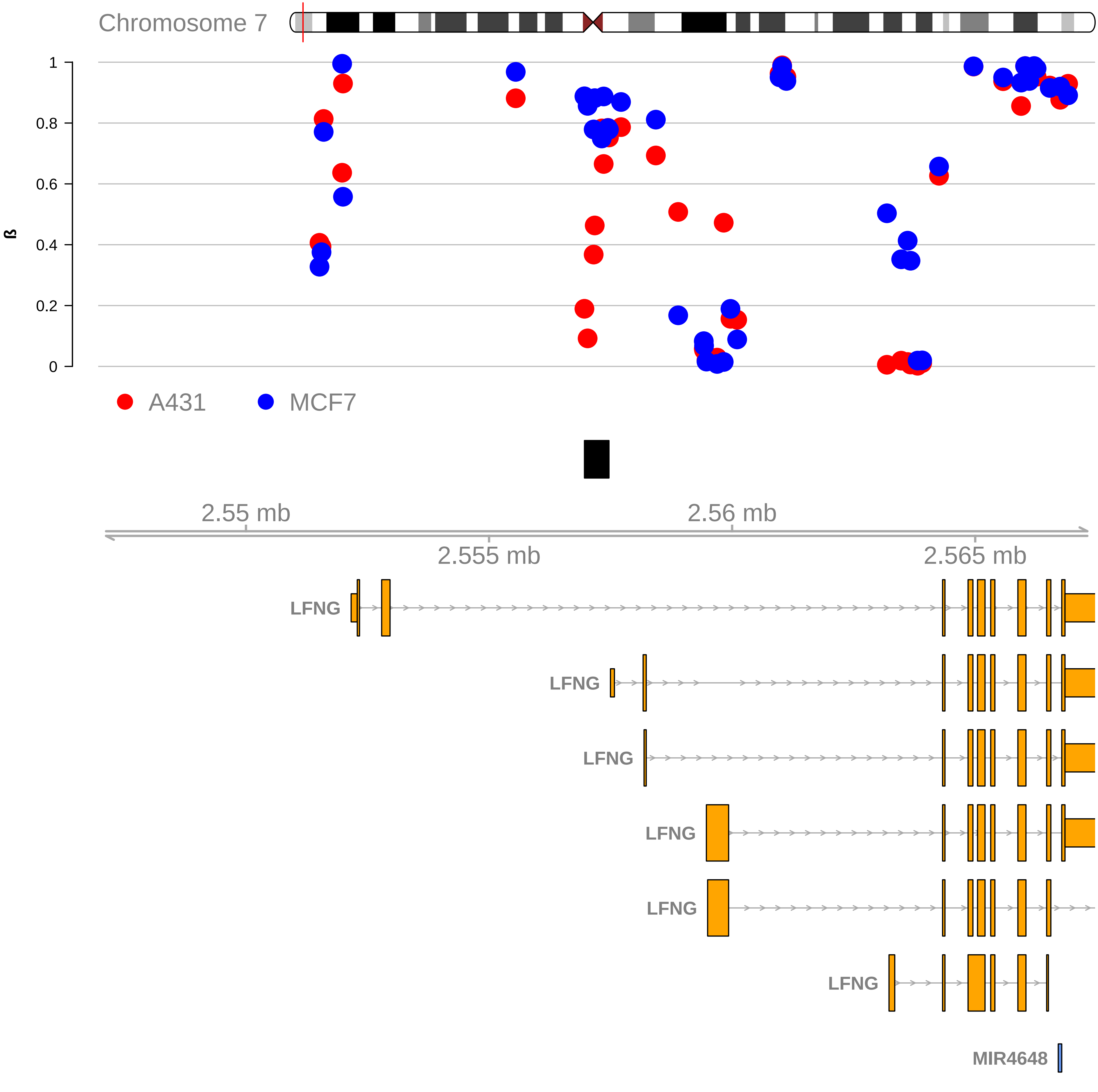

Chromosome 7
1
0.8
0.6
0.4
0.2
0
ß
A431
MCF7
2.55 mb
2.555 mb
2.56 mb
2.565 mb
LFNG
LFNG
LFNG
LFNG
LFNG
LFNG
MIR4648
